# Diseases of the Eye

**Published:** 1900-09

**Authors:** 


DISEASES OF THE EYE. By EDWARD NETTLESHIP, F. R. C. S., Ophthalmic Surgeon at St. Thomas' Hospital, London; Surgeon to the Royal London Ophthalmic Hospital, etc. New (6th) American from the sixth English edition, thoroughly revised by WILLIAM CAMPBELL POSEY, M. D. With a supplement on the detection of color blindness by WILLIAM THOMSON, M. D., Professor of Ophthalmology in the Jefferson Medical College, Philadelphia. In one 12mo volume of 562 pages, with 192 illustrations. Selections from Snellen's test types and formulæ and 5 colored plates. Philadelphia and New York: Lea Brothers & Co. [Price, cloth, $2.25 net.]

Only a short time has elapsed since the American publishers issued the fifth, from the sixth English edition of this standard manual of Nettleship. The rapid exhaustion of that edition has demanded a sixth American, which is now before us. This edition differs from all others in being revised and edited by an American ophthalmologist, Dr. Posey, of Philadelphia. A few changes and additions have been made by him which very materially enhance its value from the American standpoint of practice, notably in the section on refraction. He has also given the latest views regarding the bacteriological origin of several forms of conjunctivitis, and has described several new forms of ocular disease.

Dr. Thomson has also availed himself of this opportunity to revise his chapter on the examination of railroad employees and has made some useful additions.

This edition of "Nettleship" therefore, while preserving all the compactness and comprehensiveness of former editions, has additional matter which will assure its continued popularity in this country.

